# Genetic association study of intron variants in the forkhead box protein P3 gene in Chinese patients diagnosed with cervical cancer

**DOI:** 10.1111/jcmm.17276

**Published:** 2022-03-24

**Authors:** Feng Shi, Xiao‐xia Pang, Guang‐jing Li, Zhi‐hong Chen, Ming‐you Dong, Jun‐li Wang

**Affiliations:** ^1^ Reproductive Medicine Center The Affiliated Hospital of Youjiang Medical University for Nationalities Baise Guangxi China; ^2^ Blood Transfusion Department The Affiliated Hospital of Youjiang Medical University for Nationalities Baise Guangxi China; ^3^ Basic Medical College Youjiang Medical University for Nationalities Baise Guangxi China; ^4^ Medical Laboratory College Youjiang Medical University for Nationalities Baise Guangxi China

**Keywords:** cervical cancer, forkhead box protein P3, genetic variants, human papillomavirus, immune infiltration

## Abstract

The aim of this study was to investigate the effects of forkhead box protein P3 (FOXP3) intron single nucleotide variants (SNVs) in high‐risk human papilloma virus (HR‐HPV) infection and cervical cancer (CC) malignant lesions. We performed FOXP3 genotyping in 350 patients with CC and 350 healthy controls using the ImLDR multiple single nucleotide polymorphism genotyping technology. The heterozygous mutation TC in rs2294021 decreased the risk of HR‐HPV infection and CC malignant lesions (TC vs. TT: OR = 0.71, 95% CI = 0.51–0.99); the dominant model TC+CC and allele C in rs2294021 decreased the risk of CC malignant lesions (TC+CC vs. TT: OR = 0.69, 95% CI = 0.50–0.95; C vs. T: OR = 0.78, 95% CI = 0.63–0.97). The heterozygous mutation GA, dominant model GA+AA and allele A in rs3761549 also decreased the risk of HR‐HPV infection and CC malignant lesions (GA vs. GG: OR = 0.70, 95% CI = 0.51–0.96; GA+AA vs. GG: OR = 0.69, 95% CI = 0.51–0.94; A vs. G: OR = 0.75, 95% CI = 0.58–0.96). Patients with CC and HR‐HPV infection carrying rs2294021 TC and rs3761549 GA had lower expression of FOXP3 protein. Haplotype analysis revealed that T‐C‐A decreased the risk of HR‐HPV infection. Furthermore, we found a significant association between immune cells infiltration and prognosis in patients with CC. Our findings demonstrated that rs2294021 and rs3761549 variants may protect against HR‐HPV and CC malignant lesions by downregulating FOXP3 and that FOXP3 was associated with immune cells infiltration, which affected the prognosis of CC.

## 1 INTRODUCTION

The incidence of cervical cancer (CC) recently decreased from 8.2% in 2008 to 7.5% in 2018.^1^ However, in 2018, approximately 570,000 new cases of CC were reported, and 311,000 CC‐related deaths occurred.^2^ CC is the fourth most common cancer in women worldwide, surpassed only by breast cancer, colorectal cancer and lung cancer, and is therefore a serious threat to the physical and mental health of women. Moreover, the cumulative incidence and mortality of CC in developing countries are 2–4 times higher than those in developed countries, particularly in lower‐resource countries. The highest number of CC cases was reported in China (106,000 cases), whereas the highest number of CC‐related deaths was reported in India (60,000 cases); furthermore, China and India together accounted for 35% of cases and deaths globally worldwide.^2^

High‐risk human papillomavirus (HR‐HPV) infection is an important cause of CC, and 99.7% of patients with CC carry this virus.^3^ In the initial stage of infection, patients may have no obvious clinical manifestations. However, persistent HPV infection is the main cause of high‐grade squamous intraepithelial lesions of the cervical epithelium and CC. Furthermore, most HPV subtypes can be cleared by the immune system, and only approximately 10% of HPV‐infected individuals will subsequently develop into precancerous lesions and CC.^4^ Nevertheless, HPV infection alone is not sufficient to induce CC onset; smoking, oral contraceptives and multiple sexual partners are also important factors affecting the development of CC.^5^ The susceptibility to and pathogenesis of CC are associated with persistent HPV infection and host‐reaction interactions. Thus, host genetic variants may affect the development of CC.^6^ Indeed, growing evidence has indicated that variants of methylenetetrahydrofolate reductase, Toll‐like receptor (TLR) 4 and TLR9 are closely associated with the risk of CC.^7^, ^8^ Therefore, further studies of the relationships between genetic variants and the pathogenesis of CC are warranted.

The forkhead box protein 3 (*FOXP3*) gene is located on the X chromosome at Xp11.23 and encodes the *FOXP3* protein, a member of the forkhead lineage‐transcription factor family. *FOXP3* is characteristically expressed in regulatory T cells (Tregs) and is involved in the activation, proliferation, differentiation and regulation of Tregs.^9^ As an important component of the tumour microenvironment, Tregs are responsible for the downregulation of autoimmune reactions and promotion of immunological tolerance. Recent studies on the roles of Tregs in tumours have demonstrated that the regulatory mechanisms mediated by Tregs contribute to immune evasion against tumour immunotherapy.^10^ Therefore, *FOXP3* is an essential factor that induces and maintains the unique immunosuppressive properties of Tregs.

Single nucleotide variants (SNVs) are the most common type of genetic mutation, and variants in the promoter region of a gene can lead to abnormal recognition by RNA polymerase Ⅱ, leading to abnormal gene expression. Furthermore, variants in the intron region of a gene may affect mRNA splicing.^11^ Importantly, *FOXP3* is regarded as tumour initiation in CC cells,^12^ and variants in the *FOXP3* gene have been shown to be associated with HPV infection and precancerous lesions.^13^ However, the relationships between *FOXP3* gene variants and the risk of persistent HR‐HPV infection and CC remain unclear.

Accordingly, in this study, we evaluated the effects of *FOXP3* variants on the occurrence of CC among Chinese individuals. In addition, we explored the role of immune‐infiltrating cells in the prognosis of patients with CC.

## 2 MATERIALS AND METHODS

### 2.1 Study design and population

In total, 350 patients with CC diagnosed at the Affiliated Cancer Hospital of Guangxi Medical University and the Affiliated Hospital of YouJiang Medical University from 2018 to 2019 were randomly enrolled as the case group, and 350 age‐matched healthy women were randomly enrolled as the control group. Before sample collection, all participants were informed of the purpose of the study and provided written informed consent for participation in the study. The inclusion criteria were as follows: all patients were diagnosed with CC, and the diagnosis was confirmed pathologically. The exclusion criteria were as follows: patients who had a history of radiotherapy and chemotherapy for treatment or a history of immune diseases or other cancers. The control participants had no history of immune disease, cervical lesions or HPV infection. The study was approved by the Ethics Committee of the Affiliated Hospital of YouJiang Medical University for Nationalities.

### 2.2 Sample collection

Cervical epithelial cytology samples were collected using cytobrushes and stored in 2 ml TE buffer at −4℃ until HPV detection and genotyping once a week. Peripheral blood was collected with EDTA2+ anticoagulant and stored at −80℃ until *FOXP3* gene variants genotyping. In addition, serum was separated from peripheral blood at 2500 *g* for 10 min and stored at −80℃ until analysis of serum *FOXP3* protein levels.

### 2.3 HPV DNA detection and genotyping

Genomic DNA for HPV DNA detection and genotyping was extracted from cytobrushes using DNAzol (YaNeng Bioscience) according to the manufacturer's instructions, and HPV subtypes were then genotyped by polymerase chain reaction (PCR)‐reverse dot blot. The reaction conditions were as follows: 25 µl reaction volume containing primers, dNTPs, Taq DNA polymerase, buffer, UNG enzyme and 5 µl HPV DNA; annealing temperature, 50°C. For the HPV DNA amplification reaction, we used HPV16 DNA as the positive control and a sample without HPV DNA as the negative control to exclude the possibility of contamination. The PCR conditions were as follows: 50°C for 15 min; 95°C for 10 min; 40 cycles of 94°C for 30 s, 42°C for 90 s and 72°C for 30 s; and 72°C for 5 min. The HPV genotype was identified with a blue dot and then was classified into six low‐risk types (HPV6, ‐11, ‐42, ‐43, ‐81 and ‐83) and 17 high‐risk types (HPV16, ‐18, ‐31, ‐33, ‐35, ‐39, ‐45, ‐51, ‐52, ‐53, ‐56, ‐58, ‐59, ‐66, ‐68, ‐73 and ‐82).

### 2.4 Genomic DNA extraction and *FOXP3* genetic genotyping

Genomic DNA for *FOXP3* variants genotyping was extracted from peripheral blood using a TIANGEN kit according to the manufacturer's guidelines. ImLDR multiple single nucleotide polymorphism genotyping technology was performed to detect rs2280883, rs2294021 and rs3761549 in the *FOXP3* gene. For rs2280883 and rs2294021, thymine (T) was replaced with cytosine (C), whereas in rs3761549, guanine (G) was replaced with adenine (A). The amplified primer sequences for *FOXP3* gene variants were designed using Primer software 3.0, as follows: rs2280883, 5′‐GAAGGAGTTGGGATGGGGTGAT‐3′ (forward) and 5′‐CCATCTCTGCACCTTGCCCTAA‐3′ (reverse); rs2294021, 5′‐ACACATGAGG ACCCTCCACTGC‐3′ (forward) and 5′‐CCCAGCCAGCCAATTAGCAGAT‐3′ (reverse); and rs3761549, 5′‐CGACACCACGGAGGAA GAGAAG‐3′ (forward) and 5′‐CAAACCTGGGTCCTCTCCACAA‐3′ (reverse). The reaction contained 1 µl DNA sample, 0.3 mM dNTPs, 1 µl primers for multiple PCR, 1 µl HotStarTaq polymerase (Qiagen Inc.), 3.0 mM Mg^2+^ and 1× HotStarTaq buffer. The volume was brought to 20 µl using ultrapure water. The PCR conditions were as follows: denaturation at 95°C for 2 min; 11 cycles of 94°C for 20 s, 65°C for 40 s, and 72°C for 90 s; 24 cycles of 94°C for 20 s, 59°C for 30 s and 72°C for 90 s; elongation at 72°C for 2 min; and final cooling at 4°C. After amplification, the PCR products were purified by incubation with shrimp alkaline phosphatase (Promega) and exonuclease I (Epicentre Company) at 37°C for 60 min and 75°C for 15 min. Then, the reaction of allele‐specific multiplexed ligase was performed with 2 µl PCR product, 0.4 µl of 1 µM 5′ primer, 0.4 µl of 2 µM primer, 0.25 µl thermostable ligase, 1 µl of 10× buffer and 6 µl ddH_2_O for 35 cycles of 94℃ for 1 min and 56℃ for 4 min. Finally, variants in the *FOXP3* gene were identified by capillary electrophoresis.

### 2.5 Serum *FOXP3* detection

Serum *FOXP3* protein levels were detected using an enzyme‐linked immunosorbent assay (ELISA) kit (eBioscience) according to the manufacturer’s instructions. The absorbance value was measured using an enzyme‐labelled metre at 450 nm (RT‐6000, China), and the concentration of serum *FOXP3* protein was analysed using a standard curve.

### 2.6 Immune cells infiltration and prognosis in patients with CC

To reliably evaluate immune cells infiltration in CC, we used CIBERSORT to detect the relative proportions of immune cells in each CC sample. An algorithm with 1000 permutations was adopted to obtain meaningful results, and the expression of immune checkpoint‐related genes and infiltrating levels of immune cells in high and low *FOXP3* expression groups of patients with CC were analysed and drawn using the ‘limma’ and ‘ggplot2’ packages in R respectively. Survival curves were drawn for each type of immune cells using the ‘survminer’ and ‘survival’ packages in R. In addition, the CIBERSORT, CIBERSORT‐ABS, MCPcounter, XCELL, EPIC, QUANTISEQ and TIMER algorithms were compared with assess cellular components or cell immune responses between high‐*FOXP3* expression and low‐*FOXP3* expression groups. The differences in immune responses under different algorithms were determined using a heatmap.

### 2.7 Statistical analysis

Pearson’s chi‐square (*χ* ^2^) test was used to analyse differences in sociodemographic and clinical characteristics and *FOXP3* gene variants between the case and control groups. The Hardy–Weinberg equilibrium was tested using Pearson’s chi‐square test. Binary logistic regression was used to analyse the frequency distribution differences of genotypes and alleles among the HR‐HPV‐positive, HR‐HPV‐negative and control groups. Haplotype analysis was performed using online SHEsis software (http://analysis.bio‐x.cn/myAnalysis.php). The odds ratios (ORs) and 95% confidence intervals (95% CIs) were adjusted for age, smoking status, age at first sexual intercourse, history of abortion and contraceptive use. The expression of *FOXP3* protein was analysed using GraphPad Prism 5 (GraphPad Software, Inc.). Spearman's correlation was used to analyse the association between *FOXP3* expression and immune cells infiltration. The survival time according to immune infiltration status was analysed using the Kaplan–Meier method. Statistical analysis was performed using SPSS Statistics 22.0 and R 3.6.0.

## 3 RESULTS

### 3.1 Basic sociodemographic and clinical characteristics

**FIGURE 1:**
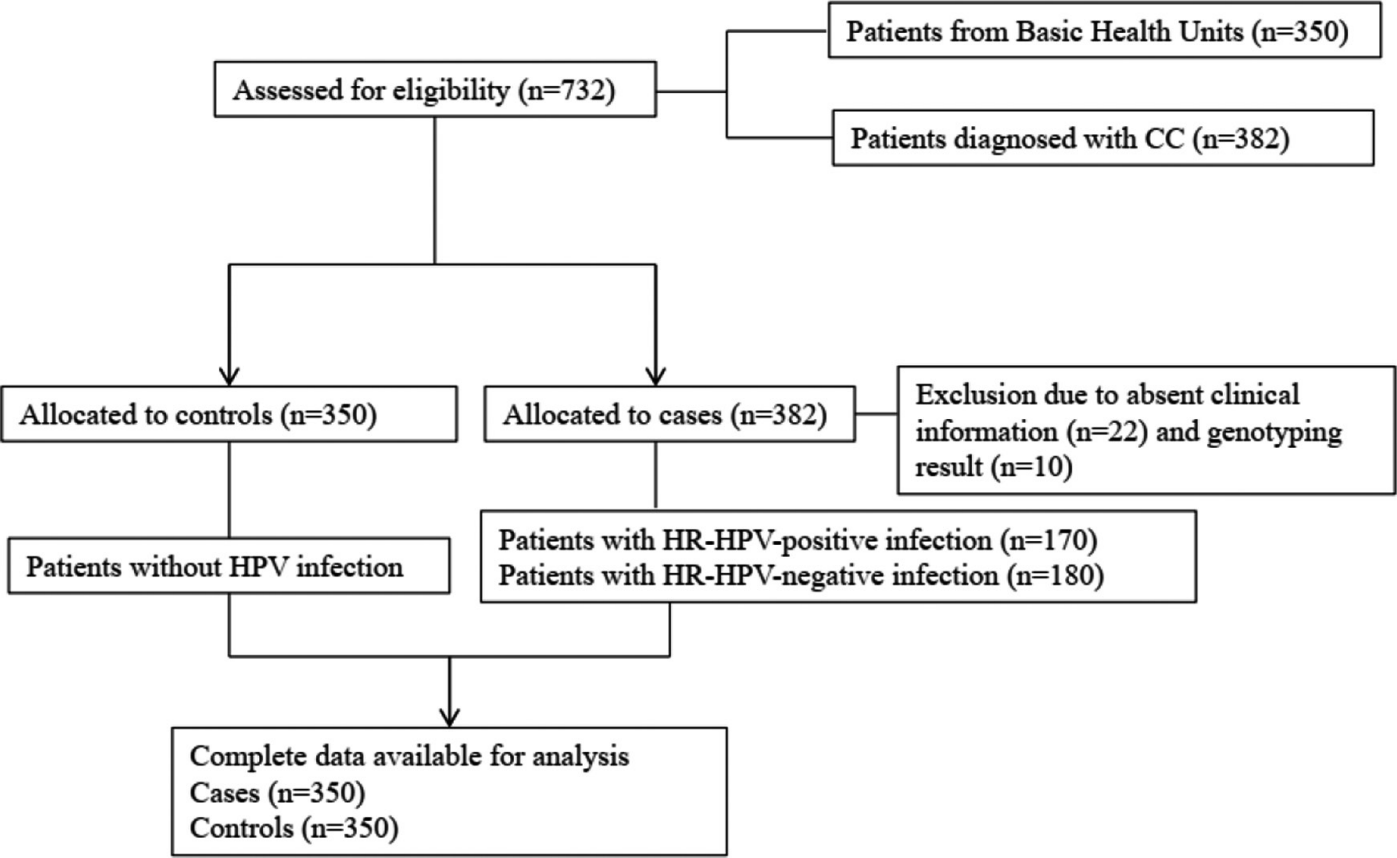
Flow of participants through the case and control study in line with the STROBE (Strengthening the Reporting of Observational Studies in Epidemiology)

**FIGURE 2:**
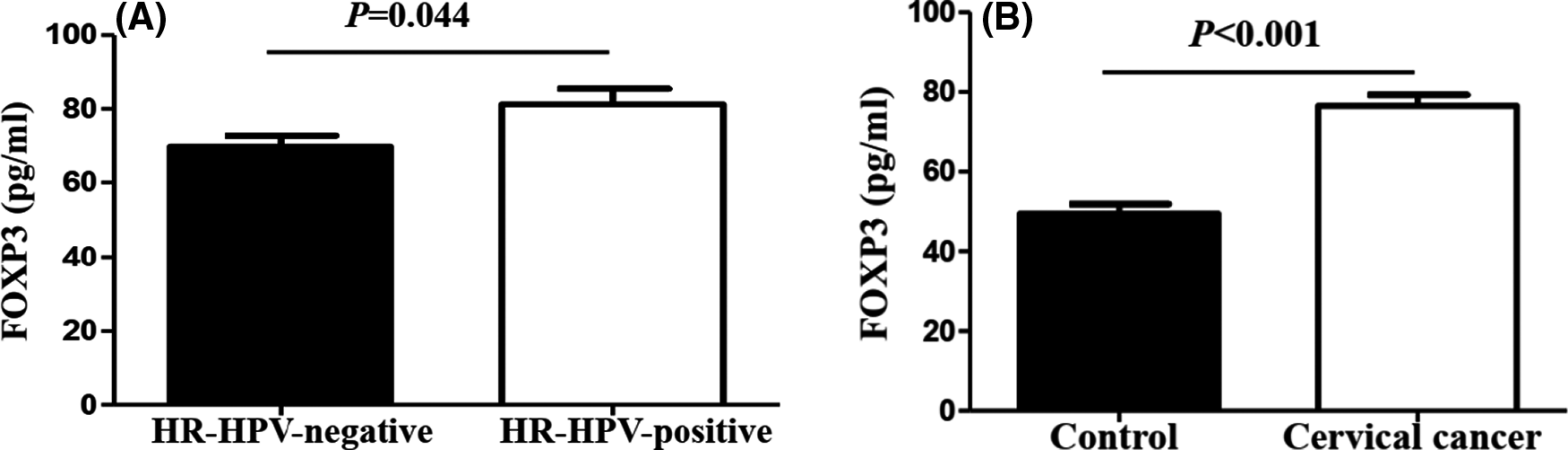
ELISA detection of serum *FOXP3* protein expression. (A) Serum levels of *FOXP3* in HR‐HPV‐negative and HR‐HPV‐positive groups. (B) Serum levels of *FOXP3* in control and CC groups

In this study, 700 women (350 patients with CC and 350 healthy individuals) were recruited. The flow chart of all subject selection through each stage of the analysis was presented in Figure 1. As shown in Table 1, the mean ages of patients with CC and control individuals were 48.07 ± 6.75 and 47.82 ± 9.50 years, respectively; there was no significant difference (*p* = 0.690). However, women with an age at first intercourse of <18 years (*p* = 0.002) and who had a history of abortion (*p* = 0.029) had a higher risk of CC. Persistent HR‐HPV infection was the main cause of CC. Thus, the CC group was divided into HR‐HPV‐positive and ‐negative groups according to HPV infection status. We found that women with an age at first intercourse of less than 18 years (*p* = 0.016) and who had a history of abortion (*p* = 0.036) were more susceptible to persistent HR‐HPV infection (Table 2). Serum *FOXP3* levels were significantly higher in the HR‐HPV‐positive and CC groups than in the HR‐HPV‐negative and control groups, respectively (*p* = 0.004 and *p* < 0.001, respectively; Figure 2).

**TABLE 1:** Basic characteristic of cervical cancer and control individuals

Characteristics	CC (*n* = 350)	Control (*n* = 350)	*p* ^†^
Age, year (mean ± SD)	48.07 ± 6.75	47.82 ± 9.50	0.690
Smoking status			
No	303 (86.6)	318 (90.9)	0.073
Yes	47 (13.4)	32 (9.1)	
Age at first sexual intercourse (year)			
<18 year	136 (38.9)	98 (28.0)	**0.002**
≥18 year	214 (61.1)	252 (72.0)	
History of abortion			
No	291 (83.1)	311 (88.9)	**0.029**
Yes	59 (16.9)	39 (11.1)	
History of contraceptive			
No	286 (81.7)	297 (84.9)	0.265
Yes	64 (18.3)	53 (15.1)	
HR‐HPV infection			
Positive	170 (48.6)		
Negative	180 (51.4)		
Tumour types			
Squamous carcinoma	275 (78.6)		
Adenocarcinoma	75 (21.4)		
Tumour stages			
Ⅰ	152 (43.4)		
Ⅱ	136 (38.9)		
Ⅲ	62 (17.7)		
Figo stages			
Ⅰ+Ⅱ	225 (64.3)		
Ⅲ+Ⅳ	125 (35.7)		
Lymph node metastasis			
Negative	257 (73.4)		
Positive	93 (26.6)		
Distant metastasis			
Negative	324 (92.6)		
Positive	26 (7.4)		

Note

There was statically significant when *p* < 0.05.

^†^ Analysed by Pearson's Chi‐square (χ^2^) test and independent‐sample *T* test.The bold value indicate that there are statistically difference (*p*<0.05).

**TABLE 2:** Basic characteristic of HR‐HPV‐positive and HR‐HPV‐negative

Characteristics	HR‐HPV‐positive (*n* = 170)	HR‐HPV‐negative (*n* = 180)	*p* ^†^
Age, year (mean ± SD)	48.24 ± 6.97	47.91 ± 6.54	0.648
Smoking status			
No	146 (85.9)	157 (87.2)	0.713
Yes	24 (14.1)	23 (12.8)	
Age at first sexual intercourse (year)			
<18 year	77 (45.3)	59 (32.8)	**0.016**
≥18 year	93 (54.7)	121 (67.2)	
History of abortion			
No	134 (78.7)	157 (87.2)	**0.036**
Yes	36 (21.2)	23 (12.8)	
History of contraceptive			
No	137 (80.6)	149 (82.8)	0.596
Yes	33 (19.4)	31 (17.2)	

Note

There was statically significant when *p* < 0.05.

^†^ Analysed by Pearson’s Chi‐square (*χ* ^2^) test and independent‐sample *T* test.

The bold value indicate that there are statistically difference *p* < 0.05.

### 3.2 *FOXP3* gene variants and susceptibility to HR‐HPV/CC

The variants of rs2280883, rs2294021 and rs3761549 in the *FOXP3* gene were selected from the 1000 Genomes Project database with minor allele frequencies >5%. The codominant model TC of rs2294021 was associated with a decreased risk of HR‐HPV infection (TC vs. TT: *p*_Adj_ = 0.023, OR = 0.59, 95% CI = 0.37–0.93). In addition, the codominant model GA, dominant model GA + AA and allele A of rs3761549 were associated with a decreased risk of HR‐HPV infection (GA vs. GG: *p*_Adj_ = 0.001, OR = 0.46, 95% CI = 0.29–0.74; GA + AA vs. GG: *p*_Adj_ = 0.002, OR = 0.49, 95% CI = 0.31–0.77; A vs. G: *p*_Adj_ = 0.009, OR = 0.61, 95% CI = 0.42–0.88; Table 3). Further investigation of *FOXP3* genetic variants and CC was performed. The results showed that patients harbouring the codominant model TC, dominant model TC + CC and allele C of rs2294021 had a lower risk of CC (TC vs. TT: *p*_Adj_ = 0.044, OR = 0.71, 95% CI = 0.51–0.99; TC + CC vs. TT: *p*_Adj_ = 0.022, OR = 0.69, 95% CI = 0.50–0.95; C vs. T: *p*_Adj_ = 0.027, OR = 0.78, 95% CI = 0.63–0.97) and those patients harbouring the codominant model GA, dominant model GA + AA and allele A of rs3761549 had a lower risk of CC (GA vs. GG: *p*_Adj_ = 0.026, OR = 0.70, 95% CI = 0.51–0.96; GA + AA vs. GG: *p*_Adj_ = 0.018, OR = 0.69, 95% CI = 0.51–0.94; A vs. G: *p*_Adj_ = 0.023, OR = 0.75, 95% CI = 0.58–0.96; Table 4).

**TABLE 3:** Associations between the genotype and allele of FOXP3 and the risk of HR‐HPV

Variants	HR‐HPV‐positive (*n* = 170)	HR‐HPV‐negative (*n* = 180)	*p*	OR (95% CI)	*p* ^†^	OR (95% CI)^†^
rs2280883						
Co‐dominant						
TT	118 (69.4)	140 (77.8)	Reference	Reference	Reference	Reference
TC	47 (27.6)	38 (21.1)	0.127	1.47 (0.90–2.40)	0.093	1.54 (0.93–2.55)
CC	5 (2.9)	2 (1.1)	0.199	2.97 (0.57–15.57)	0.173	3.22 (0.60–17.31)
Recessive						
TT + TC	165 (97.1)	178 (98.9)	Reference	Reference	Reference	Reference
CC	5 (2.9)	2 (1.1)	0.240	2.70 (0.52–14.09)	0.214	2.89 (0.54–15.45)
Dominant						
TT	118 (69.4)	140 (77.8)	Reference	Reference	Reference	Reference
TC + CC	52 (30.6)	40 (22.2)	0.077	1.54 (0.96–2.49)	0.053	1.62 (0.96–2.65)
Allele						
T	283 (83.2)	318 (88.3)	Reference	Reference	Reference	Reference
C	57 (16.8)	42 (11.7)	0.054	1.53 (0.99–2.34)	0.051	1.54 (0.99–2.37)
rs2294021						
Co‐dominant						
TT	75 (44.1)	64 (35.6)	Reference	Reference	Reference	Reference
TC	70 (41.2)	98 (54.4)	**0.032**	0.61 (0.39–0.95)	**0.023**	0.59 (0.37–0.93)
CC	25 (14.7)	18 (10.0)	0.630	1.19 (0.59–2.37)	0.784	1.10 (0.55–2.34)
Recessive						
TT + TC	145 (85.3)	162 (90.0)	Reference	Reference	Reference	Reference
CC	25 (14.7)	18 (10.0)	0.182	1.55 (0.81–2.96)	0.242	1.48 (0.77–2.86)
Dominant						
TT	75 (44.1)	64 (35.6)	Reference	Reference	Reference	Reference
TC + CC	95 (55.9)	116 (64.4)	0.102	0.70 (0.46–1.07)	0.070	0.67 (0.43–1.03)
Allele						
T	220 (64.7)	226 (62.8)	Reference	Reference	Reference	Reference
C	120 (35.3)	134 (37.2)	0.596	0.92 (0.68–1.25)	0.461	0.89 (0.65–1.22)
rs3761549						
Co‐dominant						
GG	117 (68.8)	97 (53.9)	Reference	Reference	Reference	Reference
GA	43 (25.3)	74 (41.1)	**0.002**	0.48 (0.30–0.77)	**0.001**	0.46 (0.29–0.74)
AA	10 (5.9)	9 (5.0)	0.864	0.92 (0.36–2.36)	0.501	0.72 (0.27–1.89)
Recessive						
GG + GA	160 (94.1)	171 (95.0)	Reference	Reference	Reference	Reference
AA	10 (5.9)	9 (5.0)	0.716	1.19 (0.47–2.30)	0.921	1.95 (0.37–2.47)
Dominant						
GG	117 (68.8)	97 (53.9)	Reference	Reference	Reference	Reference
GA + AA	53 (31.2)	83 (46.1)	**0.004**	0.53 (0.34–0.82)	**0.002**	0.49 (0.31–0.77)
Allele						
G	277 (81.5)	268 (74.4)	Reference	Reference	Reference	Reference
A	63 (18.5)	92 (25.6)	**0.026**	0.66 (0.46–0.95)	**0.009**	0.61 (0.42–0.88)

^†^ Adjusted by age, smoking status, age at first sexual intercourse, history of abortion and contraceptive.The bold value indicate that there are statistically difference (*p*<0.05).

**TABLE 4:** Associations between the genotype and allele of FOXP3 and the risk of CC

Variants	CC *n* = 350	Control *n* = 350	*p*	OR (95% CI)	*p* ^†^	OR (95% CI)^†^
rs2280883						
Co‐dominant						
TT	258 (73.7)	252 (72.0)	Reference	Reference	Reference	Reference
TC	85 (24.3)	92 (26.3)	0.556	0.90 (0.64–1.27)	0.586	0.91 (0.64–1.29)
CC	7 (2.0)	6 (1.7)	0.817	1.14 (0.38–3.44)	0.742	1.21 (0.39–3.70)
Recessive						
TT + TC	343 (98.0)	344 (98.3)	Reference	Reference	Reference	Reference
CC	7 (2.0)	6 (1.7)	0.780	1.17 (0.39–3.52)	0.709	1.24 (0.41–3.78)
Dominant						
TT	258 (73.7)	252 (72.0)	Reference	Reference	Reference	Reference
TC + CC	92 (26.3)	98 (72.0)	0.610	0.92 (0.66–1.28)	0.655	0.93 (0.66–1.30)
Allele						
T	601 (85.9)	596 (85.1)	Reference	Reference	Reference	Reference
C	99 (14.1)	104 (14.9)	0.704	0.94 (0.70–1.27)	0.766	0.96 (0.71–1.29)
rs2294021						
Co‐dominant						
TT	139 (39.7)	111 (31.7)	Reference	Reference	Reference	Reference
TC	168 (48.0)	187 (53.4)	**0.045**	0.72 (0.52–0.99)	**0.044**	0.71 (0.51–0.99)
CC	43 (12.3)	52 (14.9)	0.087	0.66 (0.41–1.06)	0.051	0.62 (0.38–1.01)
Recessive						
TT + TC	307 (87.7)	298 (85.1)	Reference	Reference	Reference	Reference
CC	43 (12.3)	52 (14.9)	0.321	0.80 (0.52–1.24)	0.214	0.75 (0.48–1.18)
Dominant						
TT	139 (39.7)	111 (31.7)	Reference	Reference	Reference	Reference
TC + CC	211 (60.3)	239 (68.3)	**0.027**	0.71 (0.52–0.96)	**0.022**	0.69 (0.50–0.95)
Allele						
T	446 (63.7)	409 (58.4)	Reference	Reference	Reference	Reference
C	254 (36.3)	291 (41.6)	**0.043**	0.80 (0.65–0.99)	**0.027**	0.78 (0.63–0.97)
rs3761549						
Co‐dominant						
GG	214 (61.1)	185 (52.9)	Reference	Reference	Reference	Reference
GA	117 (33.4)	143 (40.9)	**0.030**	0.71 (0.52–0.97)	**0.026**	0.70 (0.51–0.96)
AA	19 (5.4)	22 (6.3)	0.374	0.75 (0.39–1.42)	0.199	0.65 (0.34–1.26)
Recessive						
GG + GA	331 (94.6)	328 (93.7)	Reference	Reference	Reference	Reference
AA	19 (5.4)	22 (6.3)	0.629	0.86 (0.45–1.61)	0.382	0.75 (0.39–1.43)
Dominant						
GG	214 (61.1)	185 (52.9)	Reference	Reference	Reference	Reference
GA + AA	136 (38.9)	165 (47.1)	**0.027**	0.71 (0.53–0.96)	**0.018**	0.69 (0.51–0.94)
Allele						
G	545 (77.9)	513 (73.3)	Reference	Reference	Reference	Reference
A	155 (22.1)	187 (26.7)	**0.047**	0.78 (0.61–0.99)	**0.023**	0.75 (0.58–0.96)

^†^ Adjusted by age, smoking status, age at first sexual intercourse, history of abortion and contraceptive.The bold value indicate that there are statistically difference (*p*<0.05).

### 3.3 Haplotype analysis of the *FOXP3* gene

Haplotype analysis of rs2280883, rs2294021 and rs3761549 genetic variants in *FOXP3* was performed in HR‐HPV and CC. There were three possible haplotypes in the HR‐HPV group and six possible haplotypes in the CC group. We found that there were significant associations between the haplotype T‐C‐A and decreased risk of HR‐HPV infection (*p* = 0.025, OR = 0.66, 95% CI = 0.46–0.95; Table 5). However, no significant association was found between haplotype and susceptibility to CC, although there was a strong tendency for the haplotype T‐C‐A to be associated with decreased risk of CC (*p* = 0.055; Table 6).

**TABLE 5:** Haplotype distributions and the susceptibility of HR‐HPV

Haplotype	HR‐HPV‐positive *n* = 340	HR‐HPV‐negative *n* = 360	χ^2^	*p*	OR (95% CI)
T‐T‐G	220 (64.7)	226 (62.8)			
C‐C‐G	57 (16.8)	42 (11.7)	3.743	0.053	1.53 (0.99–2.34)
T‐C‐A	63 (18.5)	92 (25.5)	5.007	**0.025**	0.66 (0.46–0.95)

The bold value indicate that there are statistically difference *p* < 0.05.

**TABLE 6:** Haplotype distributions and the susceptibility of CC

Haplotype	CC *n* = 700 (%)	Control *n* = 700 (%)	χ^2^	*p*	OR (95% CI)
T‐T‐G	446 (63.7)	408 (58.3)			
C‐C‐G	99 (14.1)	101 (14.4)	0.052	0.819	0.97 (0.72–1.30)
T‐C‐A	155 (22.2)	184 (26.3)	3.674	0.055	0.79 (0.62–1.01)
C‐C‐A	0 (0.0)	2 (0.3)	–	–	–
T‐C‐G	0 (0.0)	4 (0.6)			
C‐T‐A	0 (0.0)	1 (0.1)	–	–	–

### 3.4 Associations of rs2294021 and rs3761549 with clinical characteristics

The rs2294021 and rs3761549 variants were associated with the risk of HR‐HPV and CC. However, subsequent stratification analysis to investigate the roles of rs2294021 and rs3761549 in the clinical characteristics of CC revealed no significant associations of rs2294021 and rs3761549 genotypes and alleles with tumour type, tumour stage, International Federation of Gynecology and Obstetrics stage, lymph node metastasis, and distant metastasis of CC. Notably, we did observe a trend of correlation between rs2294021 genotype and lymph node metastasis of CC (Tables 7 and 8).

**TABLE 7:** rs2294021 variants and the susceptibility of clinical characteristics in CC [*n* (%)]

rs2294021	*n*	Genotype			*p*	Allele		*p*
		TT	TC	CC		T	C	
Tumour types								
Squamous carcinoma	275	109 (39.6)	134 (48.7)	32 (11.6)		352 (64.0)	198 (36.0)	
Adenocarcinoma	75	30 (40.0)	34 (45.3)	11 (14.7)	0.747	94 (62.7)	56 (37.3)	0.763
Tumour stages								
Ⅰ	152	59 (38.8)	71 (46.7)	22 (14.5)		189 (62.2)	115 (37.8)	
Ⅱ	136	60 (44.1)	62 (45.6)	14 (10.3)	0.470	182 (66.9)	90 (33.1)	0.235
Ⅲ	62	20 (32.3)	35 (56.5)	7 (11.3)	0.430	75 (60.5)	49 (39.5)	0.745
Figo stages								
Ⅰ+Ⅱ	225	88 (39.1)	108 (48.0)	29 (12.9)		284 (63.1)	166 (36.9)	
Ⅲ+Ⅳ	125	51 (40.8)	60 (48.0)	14 (11.2)	0.885	162 (64.8)	88 (35.2)	0.656
Lymph node metastasis								
No	257	108 (42.0)	114 (44.4)	35 (13.6)		330 (64.2)	184 (35.8)	
Yes	93	31 (33.3)	54 (58.1)	8 (8.6)	0.068	116 (62.4)	70 (37.6)	0.655
Distant metastasis								
No	324	127 (39.2)	157 (48.5)	40 (12.3)		411 (63.4)	237 (36.6)	
Yes	26	12 (46.2)	11 (42.3)	3 (11.5)	0.783	35 (67.3)	17 (32.7)	0.575

**TABLE 8:** rs3761549 variants and the susceptibility of clinical characteristics in CC [*n* (%)]

rs3761549	*n*	Genotype			*p*	Allele		*p*
		GG	GA	AA		G	A	
Tumour types								
Squamous carcinoma	275	170 (61.8)	93 (33.8)	12 (4.4)		433 (78.7)	117 (21.3)	
Adenocarcinoma	75	44 (58.7)	24 (32.0)	7 (9.3)	0.242	112 (74.7)	38 (25.3)	0.288
Tumour stages								
Ⅰ	152	88 (57.9)	53 (34.9)	11 (7.2)		229 (75.3)	75 (24.7)	
Ⅱ	136	89 (65.4)	40 (29.4)	7 (5.1)	0.401	218 (80.1)	54 (19.9)	0.166
Ⅲ	62	37 (59.7)	24 (38.7)	1 (1.6)	0.190	98 (79.0)	26 (21.0)	0.413
Figo stages								
Ⅰ + Ⅱ	225	134 (59.6)	77 (34.2)	14 (6.2)		345 (76.7)	105 (23.3)	
Ⅲ + Ⅳ	125	80 (64.0)	40 (32.0)	5 (4.0)	0.574	200 (80.0)	50 (20.0)	0.309
Lymph node metastasis								
No	257	163 (63.4)	80 (31.1)	14 (5.4)		406 (79.0)	108 (21.0)	
Yes	93	51 (54.8)	37 (39.8)	5 (5.4)	0.308	139 (7.7)	47 (25.3)	0.231
Distant metastasis								
No	324	198 (61.1)	109 (33.6)	17 (5.2)		505 (77.9)	143 (22.1)	
Yes	26	16 (61.5)	8 (30.8)	2 (7.7)	0.861	40 (76.9)	12 (23.1)	0.866

### 3.5 Association between *FOXP3* gene variants and *FOXP3* levels

**FIGURE 3:**
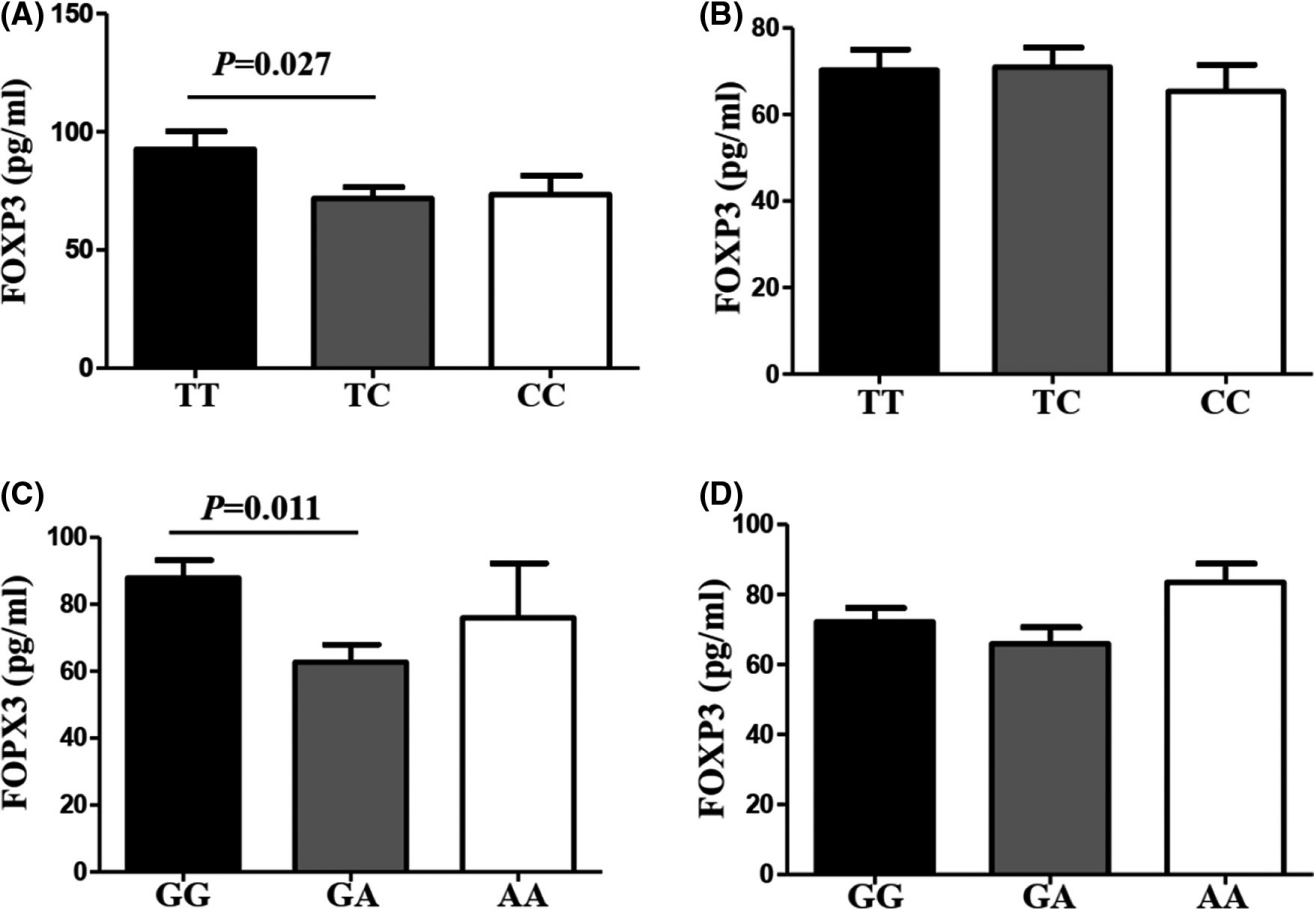
ELISA detection of serum *FOXP3* protein expression in HR‐HPV‐positive and HR‐HPV‐negative groups. (A) In HR‐HPV‐positive patients, patients harbouring genotype TC in rs2294021 had lower levels of *FOXP3* than those harbouring genotype TT. (B) There were no associations between rs2294021 variants and *FOXP3* levels in HR‐HPV‐negative patients. (C) In HR‐HPV‐positive patients, patients harbouring genotype GA in rs3761549 had lower levels of *FOXP3* than those harbouring genotype GG. (D) There were no associations between rs3761549 variants and *FOXP3* levels in HR‐HPV‐negative patients

**FIGURE 4:**
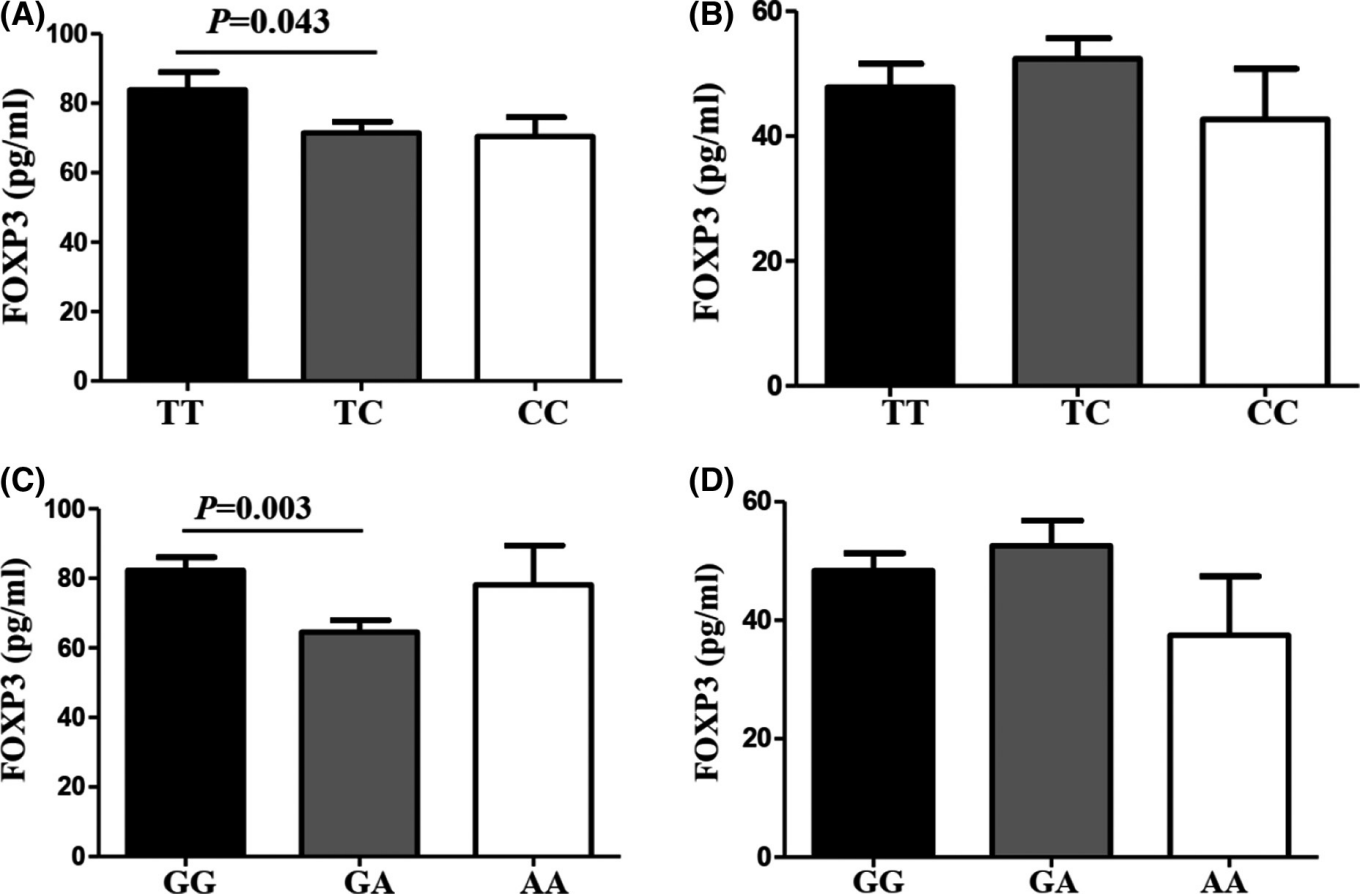
ELISA detection of serum *FOXP3* protein expression in CC and control group. (A) In the CC group, patients harbouring genotype TC in rs2294021 had lower levels of *FOXP3* than those harbouring genotype TT. (B) There were no associations between rs2294021 variants and *FOXP3* levels in the control group. (C) In the CC group, patients harbouring genotype GA in rs3761549 had lower levels of *FOXP3* than those harbouring genotype GG. (D) There were no associations between rs3761549 variants and *FOXP3* levels in the control group

The effects of *FOXP3* gene variants on serum levels of *FOXP3* were further investigated. Genotypes at rs2294021 and rs3761549 variants were significantly associated with *FOXP3* levels in patients with HR‐HPV and CC. In the HR‐HPV‐positive group, patients with genotype TC in rs2294021 and genotype GA in rs3761549 had lower levels of *FOXP3* than those with genotype TT in rs2294021 and genotype GG in rs3761549, respectively (*p* = 0.027 and *p* = 0.011, respectively; Figure 3A,C); however, there were no significant differences in serum levels of *FOXP3* among different genotypes of rs2294021 and rs3761549 in HR‐HPV‐negative patients (*p* > 0.05; Figure 3B,D). In the CC group, patients with genotype TC in rs2294021 and genotype GA in rs3761549 had lower levels of *FOXP3* than those with genotype TT in rs2294021 and genotype GG in rs3761549, respectively (*p* = 0.043 and *p* = 0.003, respectively; Figure 4A,C); however, there were no significant differences in serum levels of *FOXP3* among different genotypes of rs2294021 and rs3761549 in the control group (*p* > 0.05; Figure 4B,D).

### 3.6 Evaluation of immune cells infiltration in CC

**FIGURE 5:**
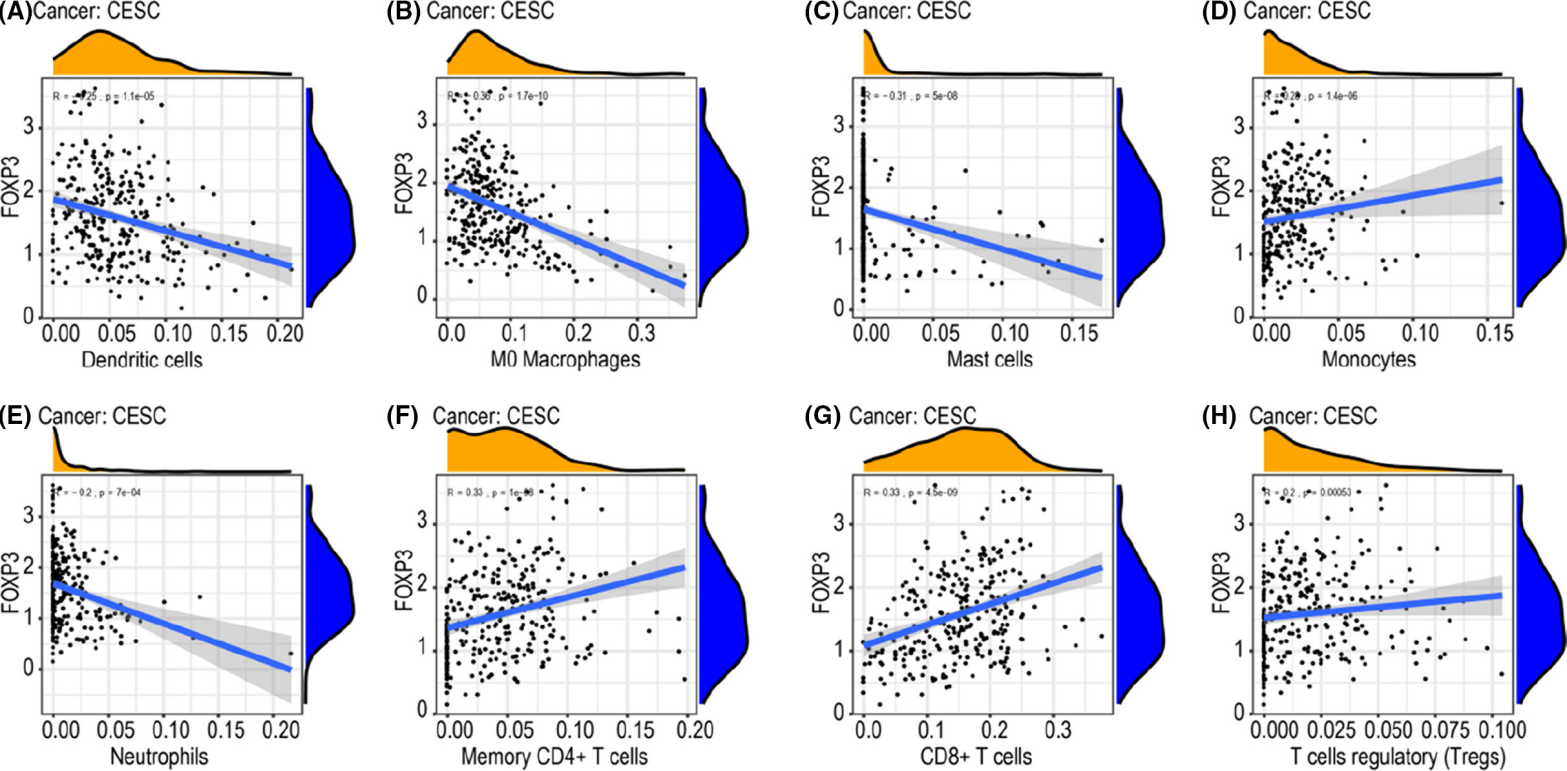
Correlation between *FOXP3* and immune cell infiltration in CC. (A) Dendritic cells. (B) M0 macrophages. (C) Mast cells. (D) Monocytes. (E) Neutrophils. (F) Memory CD4^+^ T cells. (G) CD8^+^ T cells. (H) Regulatory T cells

**FIGURE 6:**
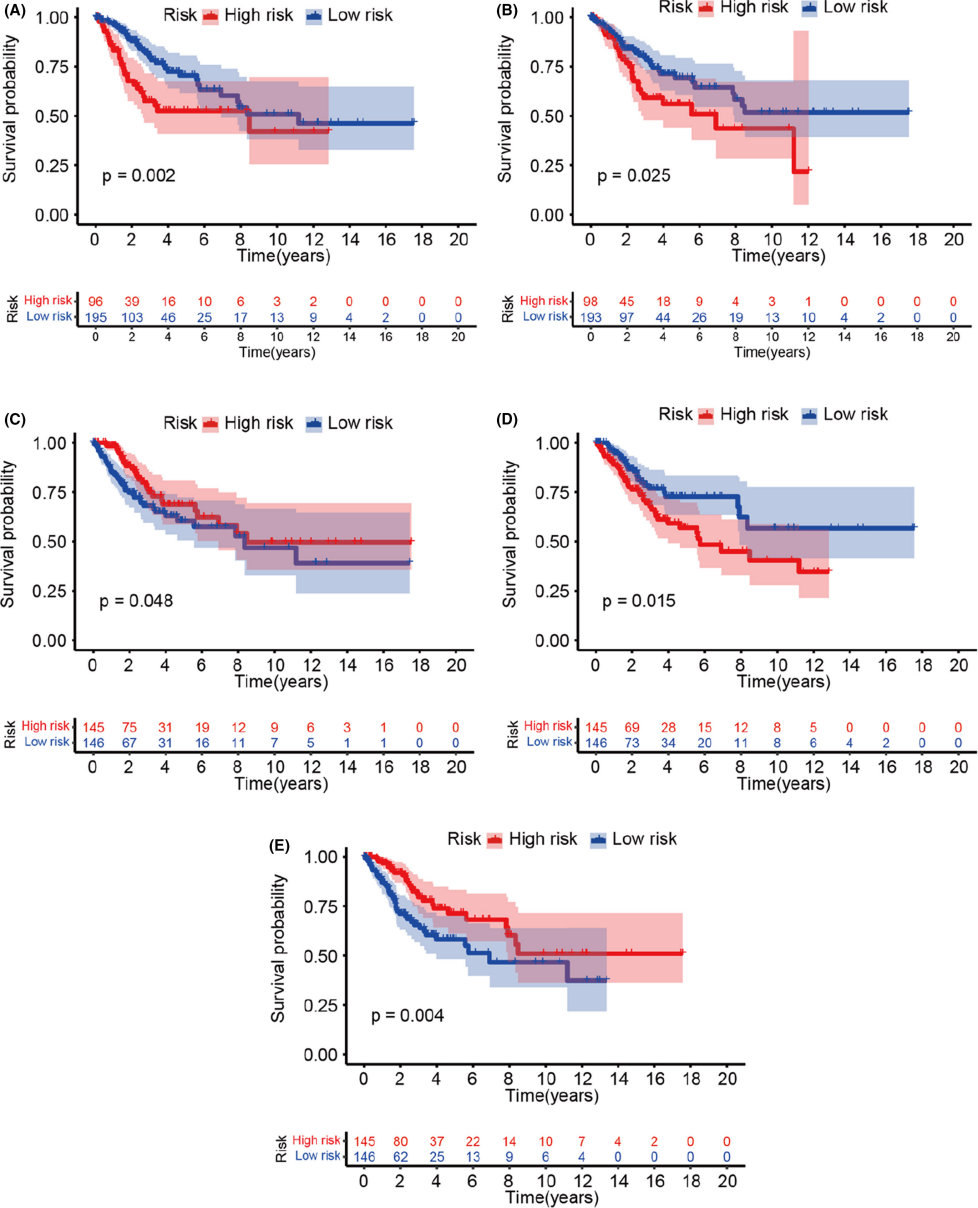
Kaplan–Meier survival analysis of patients with cervical cancer (CC) according to tumour‐infiltrating immune cell status. (A) Mast cells. (B) Neutrophils. (C) Memory CD4^+^ T cells. (D) Resting memory CD4 T cells. (E) CD8^+^ T cells

*FOXP3* participates in the activation, proliferation, differentiation and regulation of Tregs^9^ and is involved in inflammatory and immune cells infiltration. Therefore, to assess whether the immune microenvironment could be influenced by *FOXP3*, we investigated the associations of *FOXP3* expression with the levels of 22 immune cells types based on a dataset from The Cancer Genome Atlas (TCGA). As shown in Figure 5, *FOXP3* expression was significantly negatively correlated with infiltration of dendritic cells (*R* = −0.25, *p* = 1.1e‐5; Figure 5A), M0 macrophages (*R* = −0.36, *p* = 1.7e‐10; Figure 5B), mast cells (*R* = −0.31, *p* = 5e‐8; Figure 5C) and neutrophils (*R* = −0.2, *p* = 7e‐4; Figure 5E). By contrast, *FOXP3* expression was significantly positively correlated with infiltration of monocytes (*R* = 0.28, *p* = 1.4e‐6, Figure 5D), memory CD4^+^ T cells (*R* = 0.33, *p* = 1e‐8; Figure 5F), CD8^+^ T cells (*R* = 0.33, *p* = 4.5e‐9; Figure 5G) and Tregs (*R* = 0.2, *p* = 5.3e‐4; Figure 5H). Kaplan–Meier analysis revealed that mast cells (*p* = 0.002; Figure 6A), neutrophils (*p* = 0.025; Figure 6B) and resting memory CD4 T cells (*p* = 0.015; Figure 6D) were associated with poor prognosis, whereas memory CD4^+^ T cells (*p* = 0.048; Figure 6C) and CD8^+^ T cells (*p* = 0.004; Figure 6E) were protective factors in patients with CC. However, we failed to find an association between other immune cells and CC prognosis.

Next, differences in immune responses between the high‐*FOXP3* expression and low‐*FOXP3* expression groups were evaluated (Figure S1). The results also showed that groups with high *FOXP3* expression exhibited higher levels of immune cells infiltration. Finally, we analysed the expression of immune checkpoint‐related genes and infiltrating levels of immune cells in the high‐ and low‐*FOXP3* expression groups in patients with CC. As shown in Figure S2A, the expression levels of multiple immune checkpoint‐related genes were significantly different between the high‐ and low‐FOXP3 expression groups, as were the infiltration levels of resting memory CD4 T cells, memory CD4^+^ T cells, follicular helper T cells, Tregs, natural killer cells, M0 macrophages, monocytes, M1 macrophages, dendritic cells, mast cells and neutrophils (Figure S2B).

## 4 DISCUSSION

*FOXP3* was initially identified as an immune mediator involved in the differentiation and maturation of Tregs and has since been shown to play important roles in immune diseases. Furthermore, *FOXP3* is involved in antitumour immune responses and the epithelial‐mesenchymal transition in cancer^14^ and has been described in pancreatic carcinoma cells^15^ as well as many other types of cancers, including hepatocellular carcinoma, prostate cancer, breast cancer, gastric cancer, ovarian cancer and CC.^16^

In the current study, we found that women with an age at first intercourse of less than 18 years and who had a history of abortion were more prone to infection with HR‐HPV and subsequent occurrence of CC. Persistent HR‐HPV infection is a necessary but not sufficient reason for the development of CC^3^; although unhealthy social factors increase the risk of HR‐HPV, they also do not directly cause CC malignant lesions. In our current analysis, we demonstrated that the heterozygous genotype TC in rs2294021 was a good predictor of HR‐HPV infection and CC risk. Furthermore, women carrying the heterozygous genotype GA in rs3761549 were less susceptible to HR‐HPV infection and CC occurrence. In addition to CC, studies have investigated the association between *FOXP3* intron variants and other gynaecological diseases.^17^, ^18^ To the best of our knowledge, this was the first study to investigate the associations of rs2294021 and rs3761549 in the *FOXP3* gene with susceptibility to HR‐HPV infection and CC malignant lesions.

In this study, we failed to find associations of the codominant model, recessive model, dominant model and allele of rs2280883 with HR‐HPV infection and CC risk. Recently, a case–control study revealed that the rs2280883 variants, specifically the CC genotype, was significantly associated with the risk of colorectal cancer and was correlated with the clinical characteristics of lymph node metastasis and TNM stage in a Chinese population.^19^ Another study showed that patients with hepatocellular carcinoma exhibited higher frequencies of genotype TT at rs2280883 than healthy individuals.^20^ For rs2294021, we found significant associations of the codominant model TC with decreased risk of HR‐HPV infection and CC malignant lesions and of the dominant model TC+CC and allele C with decreased risk of CC malignant lesions. Furthermore, we reached similar conclusions for rs3761549. A recent case‐control study found that rs3761549 genetic variants, particularly genotype‐combined T variants, including TC, TT and TC+TT, increase the risk of Iranian patients diagnosed with brain tumours.^21^ However, another study found that the rs3761549 genotype TC decreases the risk of hepatocellular carcinoma.^20^ According to the base complementary pairing principle of DNA, base A is equal to base T and base C is equal to G. In the current study, we found that genotype A variants, including GA and GA+AA, decreased the risk of HR‐HPV infection and CC malignant lesions. Haplotype analysis of rs2280883, rs2294021 and rs3761549 revealed that the haplotype T‐C‐A decreased the risk of HR‐HPV infection and tended to be associated with reduced risk of CC malignant lesions.

*FOXP3* plays an indispensable role in preventing autoimmunity and maintaining immune stability.^22^ Dysregulation of *FOXP3* expression and alterations in *FOXP3* function affect the immune homeostasis of the host, causing autoimmune diseases and tumourigenesis.^23^, ^24^ Intron variants in genes can affect the mRNA alternative splicing process, thereby influencing the expression of corresponding proteins by disrupting transcription.^25^ Evidence from patients with CC premalignant lesions has shown that the rs3761548 genotype AA protects women from HPV infection and high‐grade squamous intraepithelial lesion development by decreasing *FOXP3* expression, whereas intron rs2232365 genotype GG increases the risk of HPV infection by promoting *FOXP3* expression.^13^ Accordingly, we deduced that rs2294021 and rs3761549 may influence *FOXP3* expression levels. Indeed, we found that serum levels of *FOXP3* were significantly upregulated in HR‐HPV‐positive and CC patients compared with HR‐HPV‐negative patients and controls. Moreover, patients with the rs2294021 TC genotype and rs3761549 GA genotype had lower levels of *FOXP3* than those with the rs2294021 TT genotype and rs3761549 GG genotype. However, there were no significant differences between rs2294021 and rs3761549 variants and *FOXP3* levels in the HR‐HPV‐negative and control groups. This phenomenon can be explained by the observation that protein expression resulting from the mRNA alternative splicing process can be regulated by variants in the intron of a gene. Thus, our results may indicate that rs2294021 and rs3761549 variants decrease the risk of HR‐HPV infection and CC malignant lesions by downregulating serum *FOXP3* levels.

The immune‐related gene *FOXP3* is an immune cytokine which mediates the development and function of Tregs. Immune cells infiltration has been shown to influence prognosis and drug resistance.^26^, ^27^ Indeed, in this study, we found negative correlations between *FOXP3* expression and the infiltration of dendritic cells, M0 macrophages, mast cells and neutrophils and positive correlations between *FOXP3* expression and the infiltration of monocytes, memory CD4^+^ T cells, CD8^+^ T cells and Tregs. Dendritic cells are specific antigen‐presenting cells that have important roles in the regulation of innate and adaptive immune responses and in antitumor immunity.^28^ One study reported that dendritic cells were positively related to prognosis in patients with glioma.^29^ In addition, mature dendritic cells have been shown to be related to favourable immune infiltrates and improved prognosis in patients with ovarian carcinoma.^30^ Macrophages are involved in the regulation of inflammation and the progression of tumourigenesis and are abundant in various cancers, including breast cancer, colorectal cancer and CC.^31^, ^32^ Moreover, relatively high levels of M0 macrophages are related to a high risk of relapse in digestive system cancers.^33^ Mast cells act in a protumourigenic manner in most cancers^34^, ^35^ and are associated with cancer prognosis. Bioinformatics analyses have demonstrated that mast cells are positively related to overall survival in patients with glioma^29^ and clear cell renal cell carcinoma.^36^ Monocytes, as precursors of macrophages and dendritic cells, mainly originate from the bone marrow and account for 10% of leukocytes in human blood.^37^ Studies have shown that high infiltration of macrophages in the tumour microenvironment is generally associated with a poor prognosis in cancer.^38^, ^39^, ^40^ Neutrophils are released from the bone marrow and account for 50–70% of leukocytes, making them the most abundant immune cells in human blood. Neutrophils have been manipulated to adapt to tumour behaviour, and high infiltration of neutrophils indicates poor prognosis in the majority of cancers.^41^ CD4^+^ and CD8^+^ T cells are considered positive prognostic factors in tumours, and during cancer progression, CD4^+^ and CD8^+^ T cells are suppressed by Tregs; a higher ratio of CD8^+^ T cells to Tregs is generally related to a favourable prognosis in cancer, whereas the reverse ratio is related to a poor prognosis.^42^ In the current study, we demonstrated that mast cells, neutrophils and resting memory CD4 T cells were associated with a poor prognosis in patients CC. By contrast, memory CD4^+^ T cells and CD8^+^ T cells were protective factors in patients with CC.

In conclusion, our current findings demonstrated that *FOXP3* rs2294021 and rs3761549 variants were associated with a decreased risk of HR‐HPV and CC malignant lesions via downregulation of *FOXP3*. Bioinformatics analyses showed that *FOXP3* was associated with immune cells infiltration, thereby affecting the prognosis of patients with CC. Although our results provided a novel insights into clinical evaluation and diagnosis, our study still have some limitations. First, the sample size was not sufficiently large. Second, all participants in this study were enrolled from hospital, suggesting potential sample selection bias. Third, our results for the associations of *FOXP3* with immune infiltration and prognosis were based on data from TCGA database, and the outcomes were not verified by in vitro or in vivo experiments. Therefore, further investigations with larger sample sizes and expanded analyses are warranted.

## CONFLICT OF INTEREST

The authors confirm that there are no conflicts of interest.

## AUTHOR CONTRIBUTIONS

**Feng Shi:** Writing – original draft (lead). **Xiao‐xia Pang:** Writing – original draft (equal). **Guang‐jing Li:** Data curation (equal). **Zhi‐hong Chen:** Resources (equal). **Ming‐you Dong:** Methodology (lead); writing – review and editing (equal). **Jun‐li Wang:** Project administration (lead); writing – review and editing (lead).

## Supporting information

Fig S1‐S2

## Data Availability

The data that support the findings of this study are available from the corresponding author upon reasonable request.
