# Supplementary material for: Genetic association study of intron variants in the forkhead box protein P3 gene in Chinese patients diagnosed with cervical cancer
Source: J Cell Mol Med. 2022 Mar 24;26(9):2658–72. doi: 10.1111/jcmm.17276 (PMC9077298; doi:10.1111/jcmm.17276)
Supplement: Supplementary file 1 — Fig S1‐S2 [file JCMM-26-2658-s001.docx]

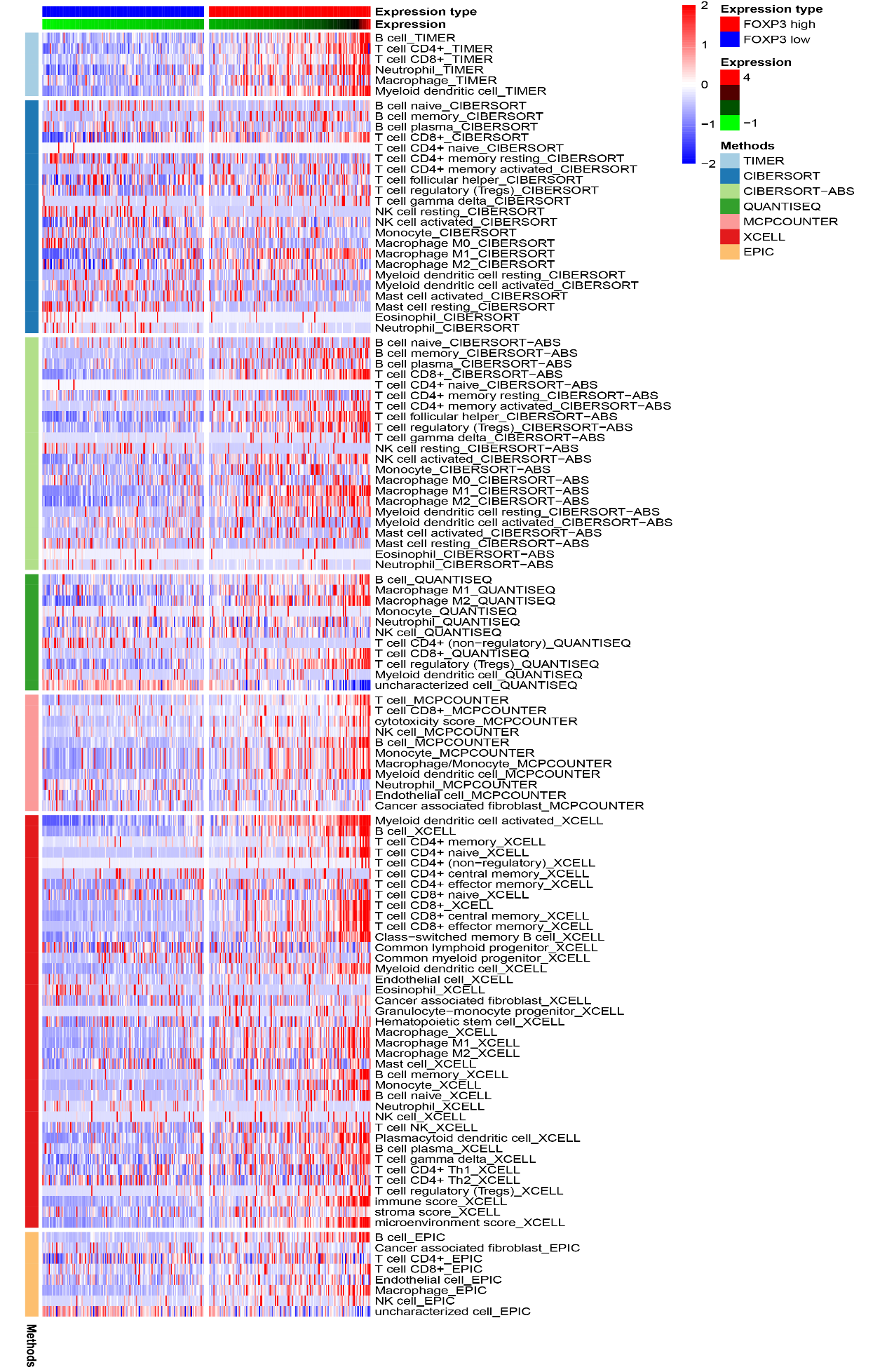


FIGURE 1 Heatmap for immune responses based on CIBERSORT, CIBERSORT-ABC, MCPcounter, QUANTISEQ, XCELL, EPIC, and TIMER algorithms between high and low *FOXP3* expression group.


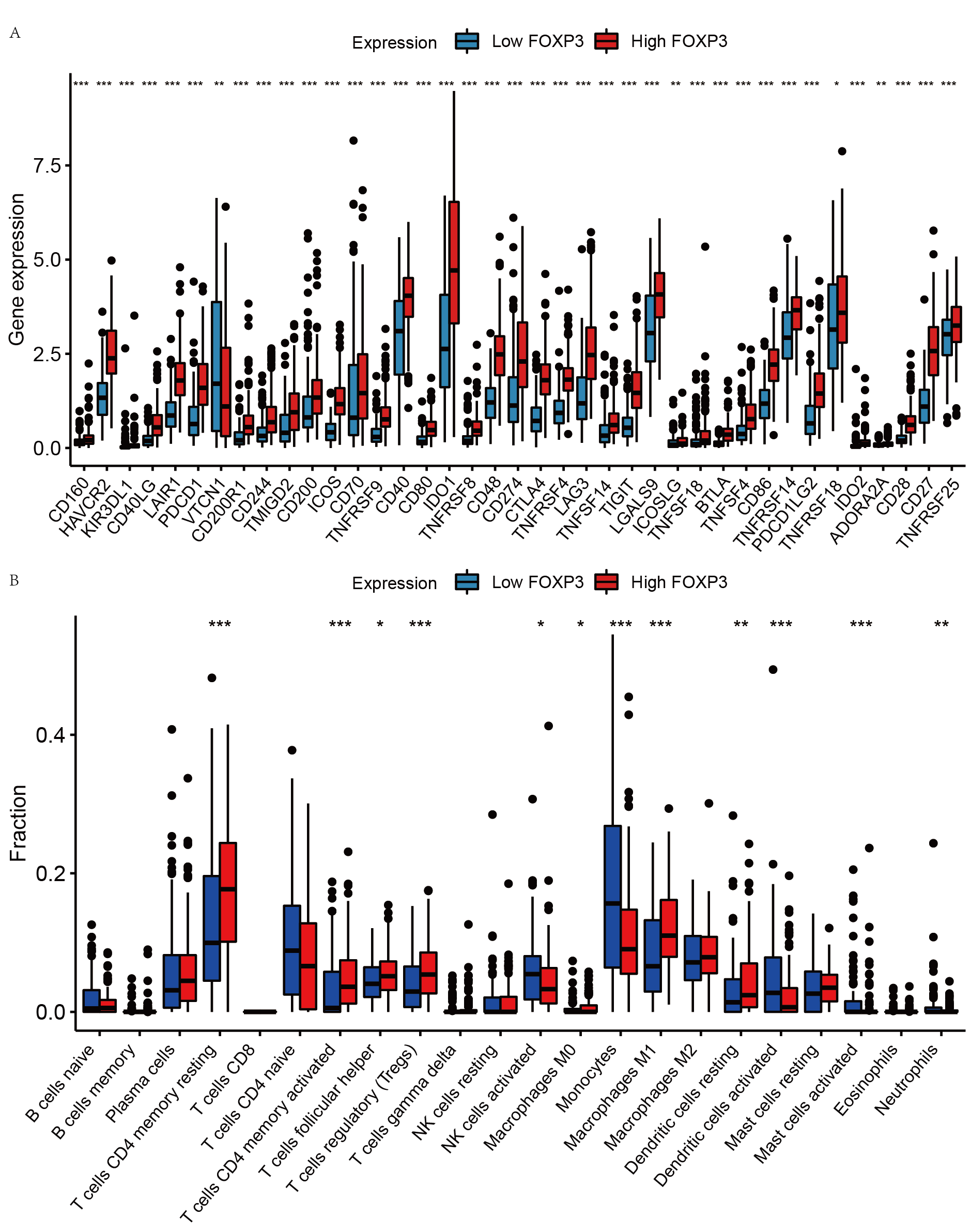


FIGURE 2 Expression of immune checkpoint-related genes and infiltrating levels of immune cells in high and low *FOXP3* expression groups in CC patients. (A: Immune checkpoint-related genes; B: Immune cells infiltrating)
